# Hepatocyte Growth Factor Prevented High-Fat Diet-Induced Obesity and Improved Insulin Resistance in Mice

**DOI:** 10.1038/s41598-017-00199-4

**Published:** 2017-03-09

**Authors:** Jun Muratsu, Masaaki Iwabayashi, Fumihiro Sanada, Yoshiaki Taniyama, Rei Otsu, Hiromi Rakugi, Ryuichi Morishita

**Affiliations:** 1 Department of Clinical Gene Therapy, Osaka University Graduate School of Medicine, Suita, Osaka 565-0871 Japan; 2 Department of Geriatric Medicine and Nephrology, Osaka University Graduate School of Medicine, Suita, Osaka 565-0871 Japan

## Abstract

Obesity and its associated chronic inflammation in adipose tissue initiate insulin resistance, which is related to several pathologies including hypertension and atherosclerosis. Previous reports demonstrated that circulating hepatocyte growth factor (HGF) level was associated with obesity and type 2 diabetes. However, its precise role in obesity and related-pathology is unclear. In this experiment, cardiac-specific over-expression of human HGF in mice (HGF-Tg mice) which showed 4–5 times higher serum HGF levels than wild-type mice were used. While body weight in wild-type mice fed with high fat diet (HFD) for 14 weeks was significantly increased accompanied with insulin resistance, HGF-Tg mice prevented body weight gain and insulin resistance. The accumulation of macrophages and elevated levels of inflammatory mediators in adipose tissue were significantly inhibited in HGF-Tg mice as compared to wild-type mice. The HFD-induced obesity in wild-type mice treated with HGF-neutralizing antibody showed an exacerbated response to the glucose tolerance test. These gain-of-function and loss-of-function studies demonstrated that the elevated HGF level induced by HFD have protective role against obesity and insulin resistance.

## Introduction

The rising prevalence of obesity is increasingly recognized as a global pandemic that threatens the health of millions of people in both developed and developing countries^1^. Obesity causes, or is closely linked to a large number of health conditions, including type 2 diabetes, hypertension, and cardiovascular disease, as well as non-metabolic derangements such as bone fragility and cancer^2–7^. Several lines of evidence support the assumption that chronic inflammation in visceral adipose tissue (but not subcutaneous adipose tissue) is the major contributor in causing insulin resistance and metabolic syndrome in the obese population^8, 9^. Excess lipid accumulation in peripheral organs such as mesenteric fat tissue, skeletal muscle, and the liver causes macrophage accumulation and inflammatory cytokine release, which devolve into a vicious cycle and impair systemic insulin sensitivity. One previous report documented that circulating hepatocyte growth factor (HGF) level was associated with the incidence of obesity and type 2 diabetes^10^. Given that HGF increases glucose uptake in adipose tissue and skeletal muscle cells^11, 12^ and promotes β cell proliferation and survival^13^, the increase in circulating HGF levels is likely to be a compensatory mechanism against insulin resistance in these patients, although its precise mechanism remains unknown. Thus, in this study, we examined whether increased serum HGF is devil or angel for obesity and insulin resistance. We previously established cardiac-specific over-expression of human HGF gene in mice (HGF-Tg mice) whose serum HGF levels are 4–5 times higher than in wild-type mice (WT). We chose cardiac specific HGF overexpression, as other strain of HGF transgenic mice such as liver and kidney specific HGF overexpression mice develop cancer and cystic diseases, which are rare in the heart^14–16^. In the present study, using HGF-Tg mice and anti-HGF neutralizing antibody (HGF-Ab), we explored the role of HGF in obese and insulin resistance induced by HFD.

## Results

### Changes in body weight in HGF-Tg mice

**Figure 1:**
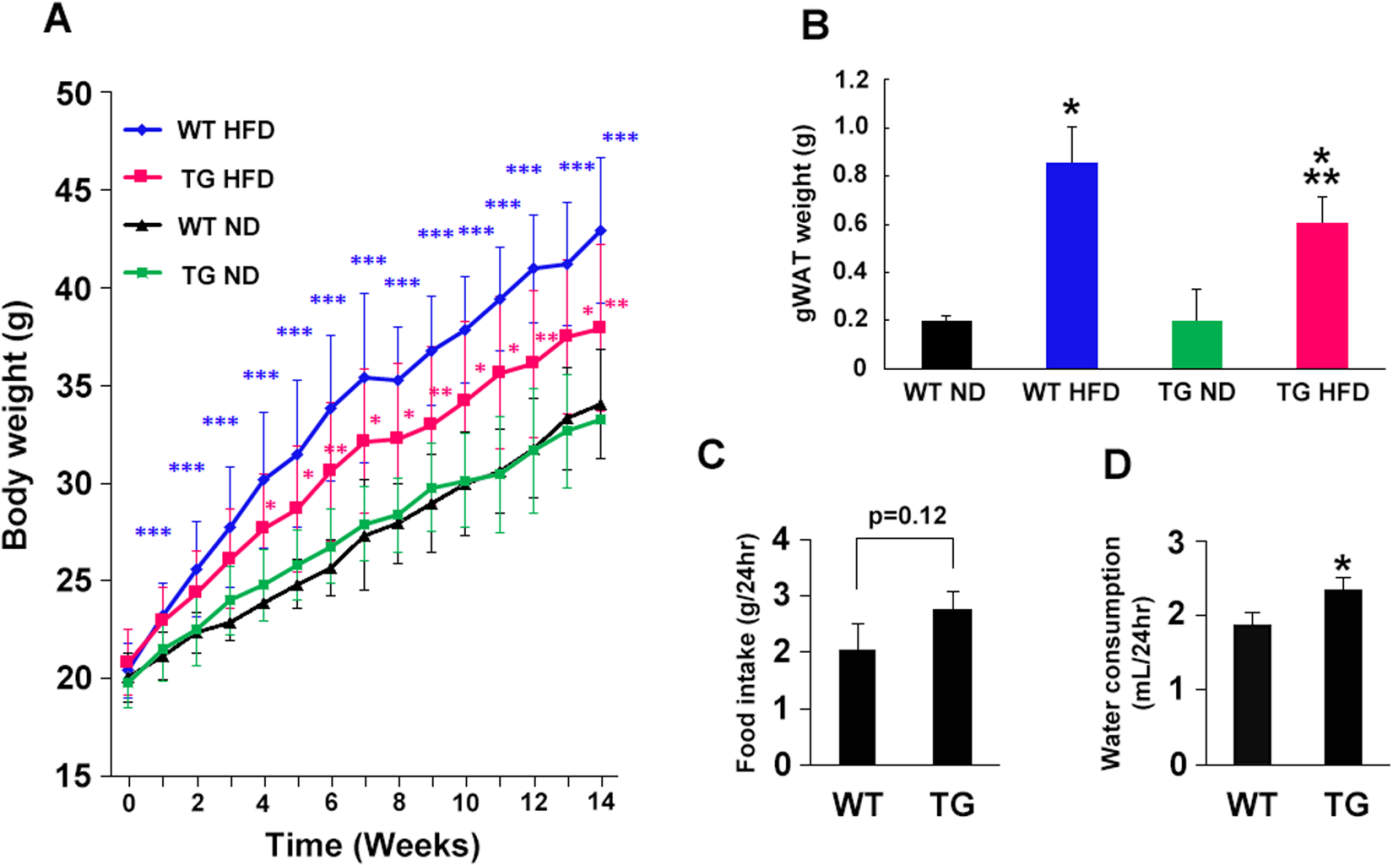
HFD-induced obesity is prevented in HGF-Tg mice. WT and HGF-Tg mice were fed a normal chow diet (ND) or HFD for 14 weeks. (**A**) Body weight was measured weekly throughout the experiment. *P < 0.05 vs. WT HFD group. **P < 0.01 vs. WT HFD group. ***P < 0.001 vs. WT ND group. N = 12–16. (**B**) Gonadal white adipose tissue (gWAT) weight. *P < 0.05 vs. WT ND and TG ND group. **P < 0.01 vs. WT HFD group. N = 5–6. (**C** and **D**) ND food intake (**C**) and water consumption (**D**) were measured in WT and HGF-Tg mice. *P < 0.05 vs. WT group. N = 4.

Body weight was measured for 14 weeks in WT and HGF-Tg mice fed with normal chow diet (ND) or high fat diet (HFD). With ND, there were no significant changes in body weight between WT and HGF-Tg mice. In contrast, body weight in HGF-Tg mice with HFD was significantly lower than those in WT mice (Fig. 1A). Similarly, increased in gonadal white adipose tissue (gWAT) weight by HFD were significantly smaller in HGF-Tg mice with HFD than those in WT mice (Fig. 1B). Intriguingly, HGF-Tg mice tended to eat more food than WT mice (Fig. 1C), and showed a significant higher level in water intake as compared to WT mice (Fig. 1D). Lipid levels such as low-density lipoprotein cholesterol, triglyceride, and free fatty acids were not different among mice, while the serum high-density lipoprotein cholesterol levels were significantly increased in HGF-Tg mice compared to WT mice following 14 weeks of HFD consumption (Figure S1).

### Glucose and insulin tolerance tests for HGF-Tg mice under ND and HFD

**Figure 2:**
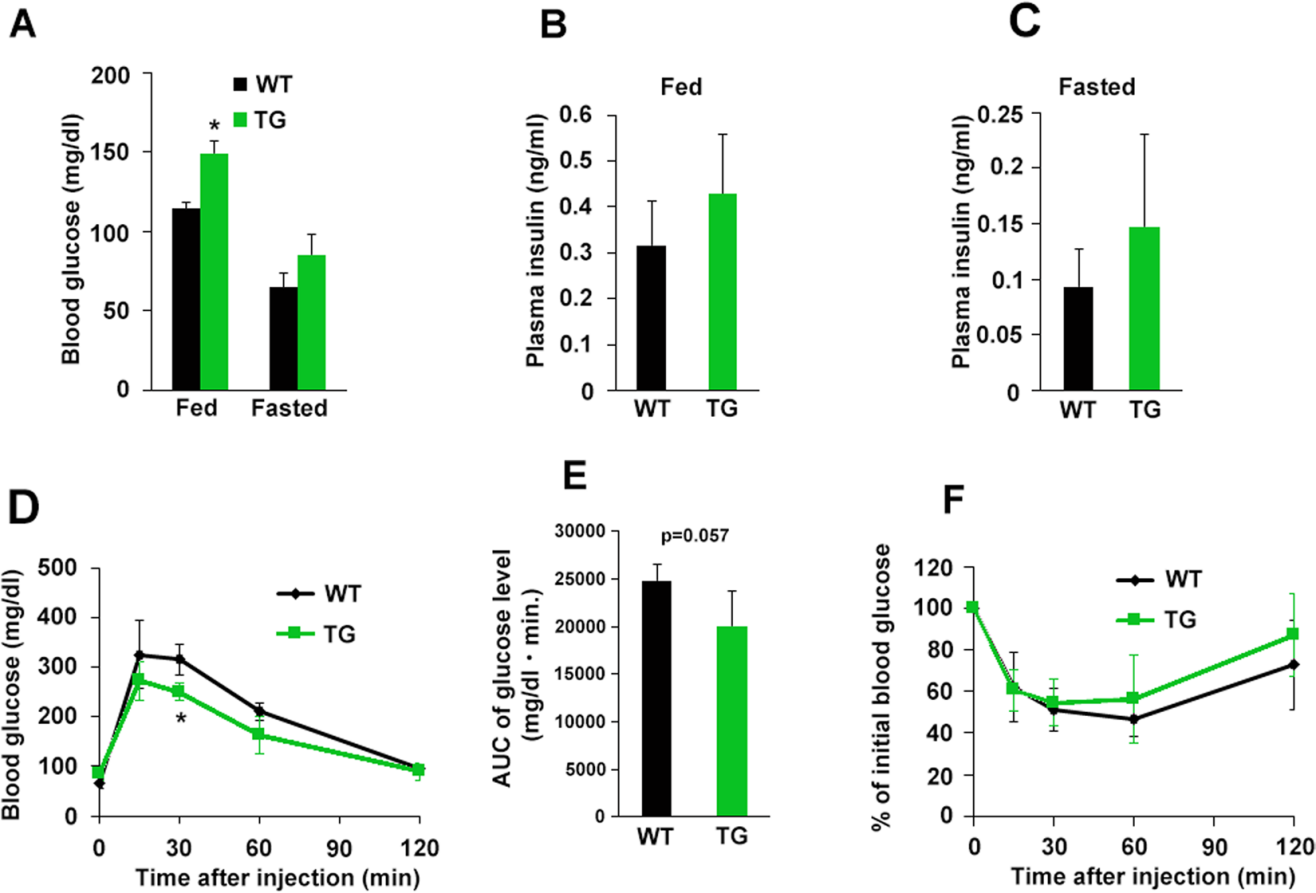
Glucose insulin tolerance of WT and HGF-Tg mice during ND consumption. (**A**–**F**) WT and HGF-Tg mice at 8 weeks of age were analyzed. (**A**–**C**) Blood glucose levels under fed conditions were significantly higher in HGF-Tg mice than in WT mice (**A**), although the plasma insulin levels were similar in both groups (**B** and **C**). *P < 0.05 vs. WT group. N = 4. (**D** and **E**) IPGTT performed in 8-week-old male WT and HGF-Tg mice. N = 4. *P < 0.05 vs. WT group. (**F**) The IPITT was comparable in WT and HGF-Tg mice fed an ND. N = 4.

To better understand the metabolic profile in HGF-Tg mice, we measured glucose and insulin levels. With ND, HGF-Tg mice exhibited slightly higher serum glucose and insulin levels (Fig. 2A–C). Despite these increased circulating glucose and insulin levels, IPGTT (2 g per kg intraperitoneally (i.p.)) showed that HGF-Tg mice were able to clear glucose from the blood at least as efficiently as WT mice (Fig. 2D and E). These data indicated that HGF-Tg mice would maintain normal glucose tolerance despite of higher basal glucose levels. In fact, when mice were injected with a fixed dose of insulin (0.75 U per kg i.p.; IPITT), HGF-Tg mice exhibited a similar glucose profile as WT mice, which is indicative of normal insulin sensitivity (Fig. 2F).

**Figure 3:**
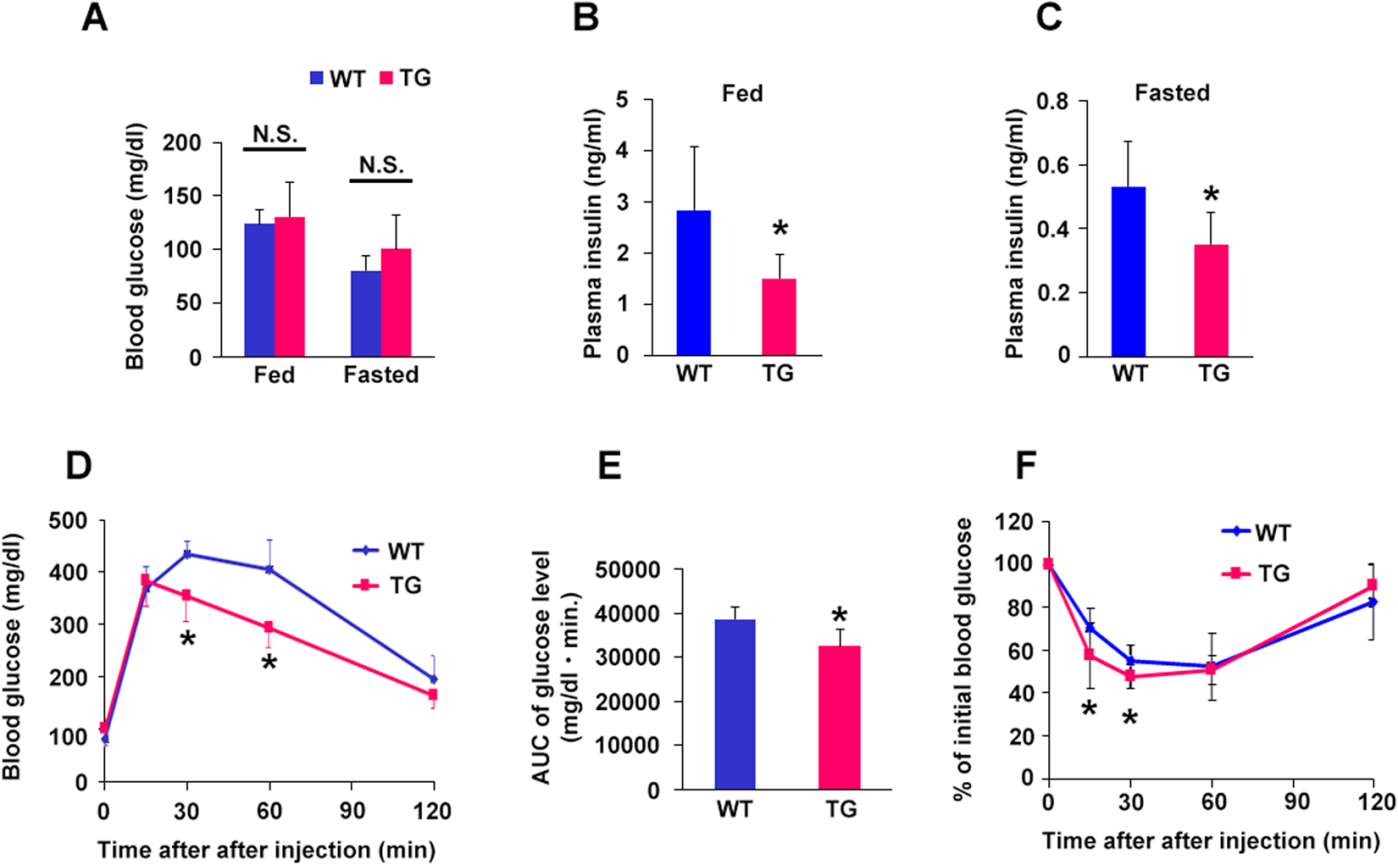
Glucose insulin tolerance of WT and HGF-Tg mice during HFD consumption. (**A**–**F**) WT and HGF-Tg mice fed an HFD were analyzed. (**A**) Although the blood glucose levels were comparable between the WT and HGF-Tg mice, the blood insulin level (**B** and **C**) was significantly lower in HGF-Tg mice than in WT mice. (**D** and **E**) IPGTT in WT and HGF-Fg mice following 14 weeks of HFD consumption. *P < 0.05 vs. WT group. N = 3–5. (**F**) IPITT was comparable in the WT and HGF-Tg mice fed an HFD. *P < 0.05 vs. WT group. N = 3–4.

On the other hand, following 14 weeks of HFD, HGF-Tg mice demonstrated lower insulin levels compared to WT mice, while serum glucose level was not differed between HGF-Tg mice and WT mice (Fig. 3A–C). IPGTT showed that HGF-Tg mice exhibited lower glucose levels at 30 and 60 minutes as compared to WT mice (Fig. 3D and E). IPITT also showed better hypoglycemic response in HGF-Tg mice, suggesting superior insulin sensitivity of insulin-responsive tissue (Fig. 3F).

### HGF neutralization by antibodies causes insulin resistance

**Figure 4:**
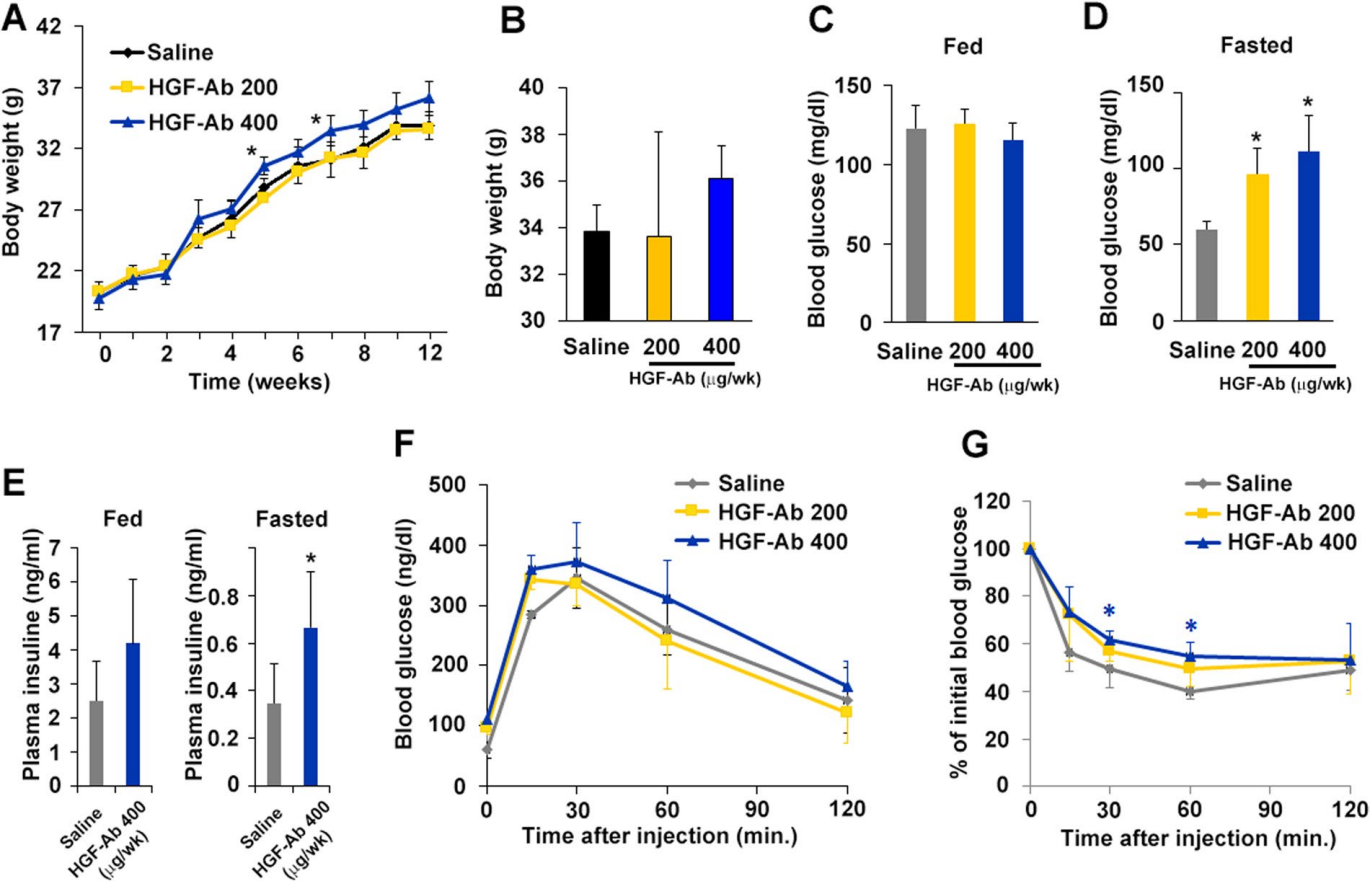
Loss of HGF function exacerbates HFD-induced insulin resistance. (**A**–**G**) Either HGF neutralizing antibody (200 or 400 µg/week) or saline was injected into WT mice fed an HFD. (**A** and **B**) Body weight was measured weekly throughout the experiment. The BW at 12 weeks is shown (**B**). *P < 0.05 vs. saline group. N = 5–6. (**C** and **D**) Blood glucose levels under fasting conditions were significantly higher in the HGF-Ab groups than the saline group. N = 4. *P < 0.05 vs. WT HFD group. (**E**) Plasma insulin levels under fasting conditions were significantly higher in the mice injected with 400 µg HGF-Ab than in saline-injected mice. *P < 0.05 vs. saline group. N = 4. (**F**) IPGTT. N = 4–6 (**G**) IPITT. N = 4–5. *P < 0.05 vs. saline group.

To further clarify the role of endogenous HGF we examined the effects of HGF neutralizing antibody (HGF-Ab) on WT mice fed with HFD, since HGF is a well-known pleiotropic cytokine originally identified as a potent mitogen for hepatocytes^17^. Until now, we and other groups have documented its angiogenic^18–20^, anti-inflammatory^21^, and anti-senescence^22^ properties. Unfortunately, although two systemic HGF knockout mouse models have been reported^23, 24^, both mice exhibited the developmental abnormalities resulting in embryonic lethality. Thus, HGF-Ab (200 µg or 400 µg) was intraperitoneally injected weekly for 12 weeks. The body weight of mice administered with 400 µg HGF-Ab was slightly increased as compared to saline group (Fig. 4A and B). Mice injected with either dose of HGF-Ab exhibited higher fasting glucose and insulin levels (Fig. 4C–E). Although no significant difference among the saline and HGF-Ab groups was observed in the IPGTT (Fig. 4F), IPITT revealed restored insulin-sensitivity in HGF-Ab 400 µg group as compared to saline group (Fig. 4G). These data indicate the existence of more pronounced insulin resistance in HGF-Ab group than saline group, which in turn implies the protective role of HGF on glucose metabolism during HFD consumption.

### HGF-Tg mice prevented HFD-induced adipose tissue inflammation

**Figure 5:**
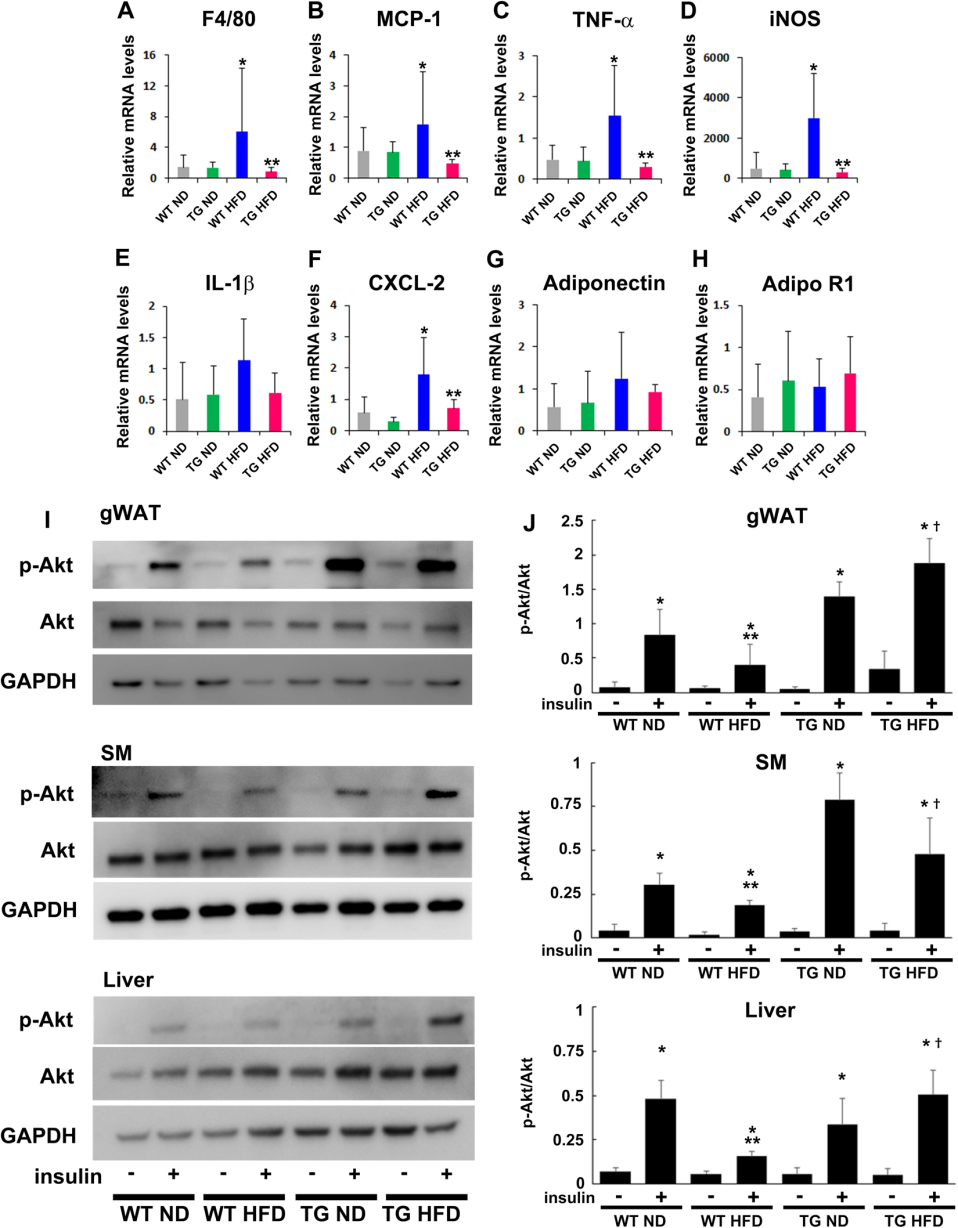
HGF inhibits adipose tissue inflammation and enhances insulin sensitivity. (**A**–**H**) The expression of inflammatory mediators in gWAT in WT mice consuming either an ND or HFD as well as WT mice treated with HGF-Tg. *P < 0.05 vs. WT ND and HGF-Tg ND groups. **P < 0.01 vs. WT HFD group. N = 6–8. (**I**,**J**) p-Akt levels of the liver, SM, and gWAT following insulin injection. *P < 0.05 vs. corresponding insulin - group, **P < 0.05 vs. WT ND insulin +group, ^†^P < 0.05 vs. WT HFD insulin +group. N = 4.

Next, we investigated the mechanism how HGF prevented HFD-induced obesity and insulin resistance. Insulin resistance in obesity arises from the combination of altered functions of insulin target cells (e.g., liver, skeletal muscle, and adipose tissue) and the accumulation of macrophages that secrete pro-inflammatory mediators in adipose tissue^25–28^. In the gWAT, the mRNA levels of the mature macrophage marker F4/80, the chemoattractants, MCP-1 and CXCL2, and the inflammatory cytokines, such as TNF-α and iNOS, were significantly increased in WT mice fed with HFD. However, these levels were markedly reduced in HGF-Tg mice fed with HFD (Fig. 5A–F). There were no significant differences in adiponectin and its receptor expression among the groups (Fig. 5G and H). Additionally, activation of Akt by insulin administration was significantly reduced in the gWAT SM, and liver by HFD; however, this activation was restored in HGF-Tg mice (Fig. 5I and J). Moreover, insulin-induced Akt signaling was decreased in HGF-Ab groups as compared to saline group under HFD condition (Figure S2). Importantly, as shown in Figure S3, HFD significantly increased the level of HGF mRNA by approximately 2 fold in gWAT, SM, and liver without changing cMet expression. All together, these data indicate that the HGF as one of the systemic gWAT, SM, and liver-derived growth factor plays a role in compensatory mechanism against insulin-resistance through the at least anti-inflammatory effect in adipose tissue.

## Discussion

The association of obesity with type 2 diabetes is well known, and the fundamental aspect of this link is the ability of obesity to induce systemic insulin resistance^25–28^. Obesity-associated insulin resistance is a major risk factor for type 2 diabetes, and is also related to a wide array of other pathophysiological abnormalities including hypertension, hyperlipidemia, atherosclerosis, and metabolic syndrome. Although there has been an explosive increase in our understanding of insulin resistance in obese individuals, many details regarding the mechanisms of what causes or improves insulin resistance remain largely unknown.

The concentration of serum HGF is elevated in obese, and is correlated with serum insulin levels and body mass index^29^. HGF secretion from the insulin target organ, such as adipocytes, liver, and skeletal muscle of obese subjects is remarkably higher than that from a healthy lean individual^30^. In this experiment using neutralizing antibody against HGF and HGF-Tg mice, we asked whether increased serum HGF is benign or malign factor for obesity and insulin resistance. We identified the following: 1) HGF expression was increased in insulin target organ, such as liver, SM, and gWAT, in WT mice fed an HFD, and inhibition of HGF by antibody exacerbated HFD-induced obesity, insulin resistance, and impaired insulin signaling. These data suggest the HGF as the systemic growth factors plays a role in insulin-resistance compensatory mechanism. However, this compensatory mechanism is insufficient to prevent adipose tissue inflammation and obesity; 2) Additional HGF over expression observed in HGF-Tg mice inhibited HFD-induced obesity and insulin resistance, accompanied by significant reduction of inflammation in gWAT. Consistently, our previous experiment has demonstrated that HGF attenuated MCP-1 and TNF-α expression of mature macrophage and inhibited hypertrophy of adipocyte in ApoE KO/HGF-Tg mice fed with HFD, which were treated with angiotensin II^31^. Thus, HGF is a strong anti-inflammatory growth factor in adipose tissue, and as a consequence, HGF could prevent HFD-induced obesity and systemic insulin resistance. Of note, HGF exerts a pleiotropic action on metabolic disorder. HGF increases glucose uptake in skeletal muscle^12^ and skeletal muscle specific HGF overexpression improved whole-body glucose tolerance independently of changes in body weight or plasma triglyceride levels through phosphorylated protein kinase B^32^. In liver, the HGF-cMet axis stimulates hepatic glucose uptake and suppresses hepatic glucose output by directly engaging insulin receptor to form a Met-Insulin receptor hybrid complex. This HGF-cMet system restores insulin responsiveness in a ob/ob mouse^33^. Interestingly, with HFD our HGF-Tg mice significantly increased FGF 21 expression in liver and serum (data not shown), which is known to increase energy expenditure through PGC-1α and browning of white adipose tissue^34^. Indeed, HGF-Tg mice showed higher insulin and glucose levels (Fig. 2A–C) with more food and water intake compared to WT mice (Fig. 1C and D), suggesting higher energy consumption. Definitely, pleiotropic action on insulin targeting organ is involved in the protective role of HGF on HFD-induced obesity and insulin resistance.

Another important perspective from this study is that certain pharmaceutical strategies that target insulin resistance could enhance HGF expression in adipose tissue, arteries, and skeletal muscle. For instance, the PPAR gamma agonists pioglitazone, irbesartan and telmisartan often used to treat patients with insulin resistance bind to and stimulate the HGF promoter to increase its expression in several organs^35–40^. Cilostazol, a PDE-3 inhibitor, improves insulin resistance^34^ and increases HGF expression through the PPAR gamma and cAMP pathway^35^. These drugs would have the potential to provide the additional beneficial properties (i.e., anti-inflammatory and anti-oxidative) through HGF production, besides their own pharmacologic targets in metabolic syndrome.

Overall, our gain-of-function study using HGF-Tg mice and loss-of-function study using anti-HGF antibody revealed that HGF prevented HFD-induced obese and systemic insulin resistance by inhibiting the inflammatory response in adipose tissue in mice. These unique character of HGF would provide us to consider new therapeutic options to treat metabolic syndrome.

## Materials and Methods

All experimental procedures were reviewed and approved by the Institutional Animal Committee at the Department of Veterinary Science of Osaka University School of Medicine and follow the recommendations of the guidelines for animal experimentation at research institutes (Ministry of Education, Culture, Sports, Science and Technology, Japan), guidelines for animal experimentation at institutes (Ministry of Health, Labor and Welfare, Japan), and guidelines for proper conduct for animal experimentation (Science Council of Japan). Male C57BL6 and HGF-Tg mice aged 8–10 weeks were used. Reagents, antibodies, and primers information are described in the online-only Data Supplement.

## Electronic supplementary material

supplement information
