# Supplementary material for: Hepatocyte Growth Factor Prevented High-Fat Diet-Induced Obesity and Improved Insulin Resistance in Mice
Source: Sci Rep. 2017 Mar 9;7:130. doi: 10.1038/s41598-017-00199-4 (PMC5427909; doi:10.1038/s41598-017-00199-4)
Supplement: Supplementary file 1 — supplement information [file 41598_2017_199_MOESM1_ESM.pdf]

Supplement information for

Hepatocyte Growth Factor Prevented High-Fat Diet-Induced Obesity and Improved Insulin Resistance in Mice

*Jun Muratsu[1, 2], *Masaaki Iwabayashi[1], *Fumihiro Sanada[1], Yoshiaki Taniyama[1, 2], Rei Otsu[1], Hiromi Rakugi[2], Ryuichi Morishita[1]

[1]Department of Clinical Gene Therapy, [2]Department of Geriatric Medicine and Nephrology, Osaka University Graduate School of Medicine, Suita, Osaka 565-0871, Japan

*These authors contributed equally to this work.

Running title; HGF against obesity and insulin resistance.

Address correspondence to:

Yoshiaki Taniyama MD, PhD, Associate Professor

Department of Clinical Gene Therapy,

Osaka University Graduate School of Medicine.

2-2 Yamada-oka, Suita, Osaka, 565-0871

Tel: +81-6-6210-8351, Fax: +81-6-6210-8359

E-mail address; taniyama@cgt.med.osaka-u.ac.jp

Ryuichi Morishita MD, PhD, Professor

E-mail: morishit@cgt.med.osaka-u.ac.jp

Key words

Obesity, Insulin resistance, Hepatocyte growth factor,

Materials and Methods

Animal Preparation

All animal procedures were performed in accordance with the guidelines of the Institutional Animal Committee at Osaka University School of Medicine. Mice with α-MHC-driven cardiac-specific over-expression of human HGF gene (HGF-Tg mice, C57BL6 background), in which the serum HGF levels were 4 to 5 times higher than those in WT mice, were used in this study. The detail characteristics of HGF-Tg mice are described in the previous report. [1] Anti-HGF neutralizing antibody (HGF-Ab) was purchased from Kringle Pharma (Osaka, Japan).

Experimental Protocol

C57/BL6 (WT) and HGF-Tg mice (all aged 8-10 weeks) were fed with normal chow diet (ND) or 1 % high-fat diet (HFD) for the indicated periods. Intraperitoneal injection of HGF-Ab (200 or 400 μg) was administered once a week throughout the experiments. Either intraperitoneal glucose tolerance test (IPGTT) or intraperitoneal insulin tolerance test (IPITT) was performed at 14 weeks following consumption of ND or HFD.

Isolation of total RNA and RT-PCR

Total RNA was isolated from mouse tissue using the RNeasy Mini Kit (QIAGEN, Hilden, Germany) according to the manufacturer's instructions. DNase-treated total RNA was reverse-transcribed with the High-Capacity cDNA Reverse Transcriptase Kit (Applied Biosystems, Foster City, CA, USA) to produce complementary DNA. The cDNA encoding the target genes was amplified and quantified by using a ViiA-7™ real-time PCR system (Applied Biosystems, Foster City, CA, USA) with the primer sets shown in Table S1.

Statistical Analysis

All statistical analyses were performed using the JMP statistics software package. Values are expressed as the means ± SE. ANOVA and t-test followed by the Tukey-Kramer adjustment for multiple comparisons were used to evaluations differences among more than two groups.

Reference

1 Sanada F, Taniyama Y, Azuma J, Iekushi K, Dosaka N, Yokoi T, Koibuchi N, Kusunoki H, Aizawa Y, Morishita R. Hepatocyte growth factor, but not vascular endothelial growth

factor, attenuates angiotensin II-induced endothelial progenitor cell senescence. Hypertension. 2009 Jan;53(1):77-82.

Figure legends

Figure S1. Blood panel from WT and HGF-Tg mice fed an ND or HFD for 14 weeks.

*P<0.01 vs. WT HFD group. N=6-8. TP; total protein, ALB; albumin, BUN; blood urea nitrogen, CRE; creatinine, AST; aspartate amino transferase, ALT; alanine amino transferase, LDH; lactate dehydrogenase, AMY; amylase, T-CHO; total choresterol, TG; triglyceride, NEFA; non-esterified fatty acids, LDL-C; low-density lipoprotein cholesterol, HDL-C; high density lipoprotein cholesterol, T-BIL; total bilirubin, T-KB; total ketone body.

Figure S2 HGF-Ab administration deteriorate insulin resistance induced by HFD.

(A) p-Akt (ser473) levels of the goandal white adipose tissue (gWAT), skeletal muscle (SM), and liver following insulin intraperitoneal injection. N=3. (B) The ratio of p-Akt/Akt. *P<0.05 vs. corresponding insulin - groups. **P<0.01 vs. HFD+HGF-Ab group. N=3.

Figure S3 HFD induces HGF and cMet expression.

(A) HGF and cMet mRNA expression of the gonadal white adipose tissue (gWAT), skeletal muscle (SM), and liver in ND or HFD-treated WT mice. N=5-6, *P<0.05 vs. WT ND.

Table S1. Primer sets used in the experiment.

	WTND	TGND	WTHFD	TGHFD
TP	5.4±0.28	6.0±0.17	5.3±0.37	5.8±0.19 *
ALB	3.6±0.18	4.1±0.16	3.4±0.20	3.8±0.19 *
BUN	29.7±6.13	28.3±2.07	24.0±3.29	23.2±2.33
CRE	0.153±0.089	0.089±0.012	0.104±0.024	0.107±0.033
AST	89.0±17.7	57.8±10.2	201.4±83.0	219.3±90.3
ALT	39.4±17.2	29.1±3.98	209.7±137.7	235.4±107.3
LDH	1021.3±572.5	525.5±455.8	1193.5±326.3	722.3±258.7 *
AMY	2655.4±290.5	2522.3±285.1	3250.4±512.4	2679.9±455.0 *
T-CHO	102.5±12.7	150.3±21.9	227.2±46.1	246.4±42.8
TG	41.9±19.2	85.5±29.0	21.1±4.68	21.3±5.00
NEFA	755.5±425.4	710.6±369.5	579.7±209.9	611.6±228.3
LCL-C	5.8±1.5	8.6±1.4	25.9±8.82	28.8±11.2
HDL-C	65.3±11.0	85.3±8.51	86.4±12.2	99.5±9.09 *
T-BIL	0.043±0.013	0.039±0.010	0.029±0.008	0.045±0.012
T-KB	609.8±459.6	291.5±239.8	538.3±309.1	598.1±362.4

Figure S1. Blood panel from WT and HGF-Tg mice fed an ND or HFD for 14 weeks.

*P<0.01 vs. WT HFD group. N=6-8. TP; total protein, ALB; albumin, BUN; blood urea nitrogen, CRE; creatinine, AST; aspartate amino transferase, ALT; alanine amino transferase, LDH; lactate dehydrogenase, AMY; amylase, T-CHO; total choresterol, TG; triglyceride, NEFA; non-esterified fatty acids, LDL-C; low-density lipoprotein cholesterol, HDL-C; high density lipoprotein cholesterol, T-BIL; total bilirubin, T-KB; total ketone body.

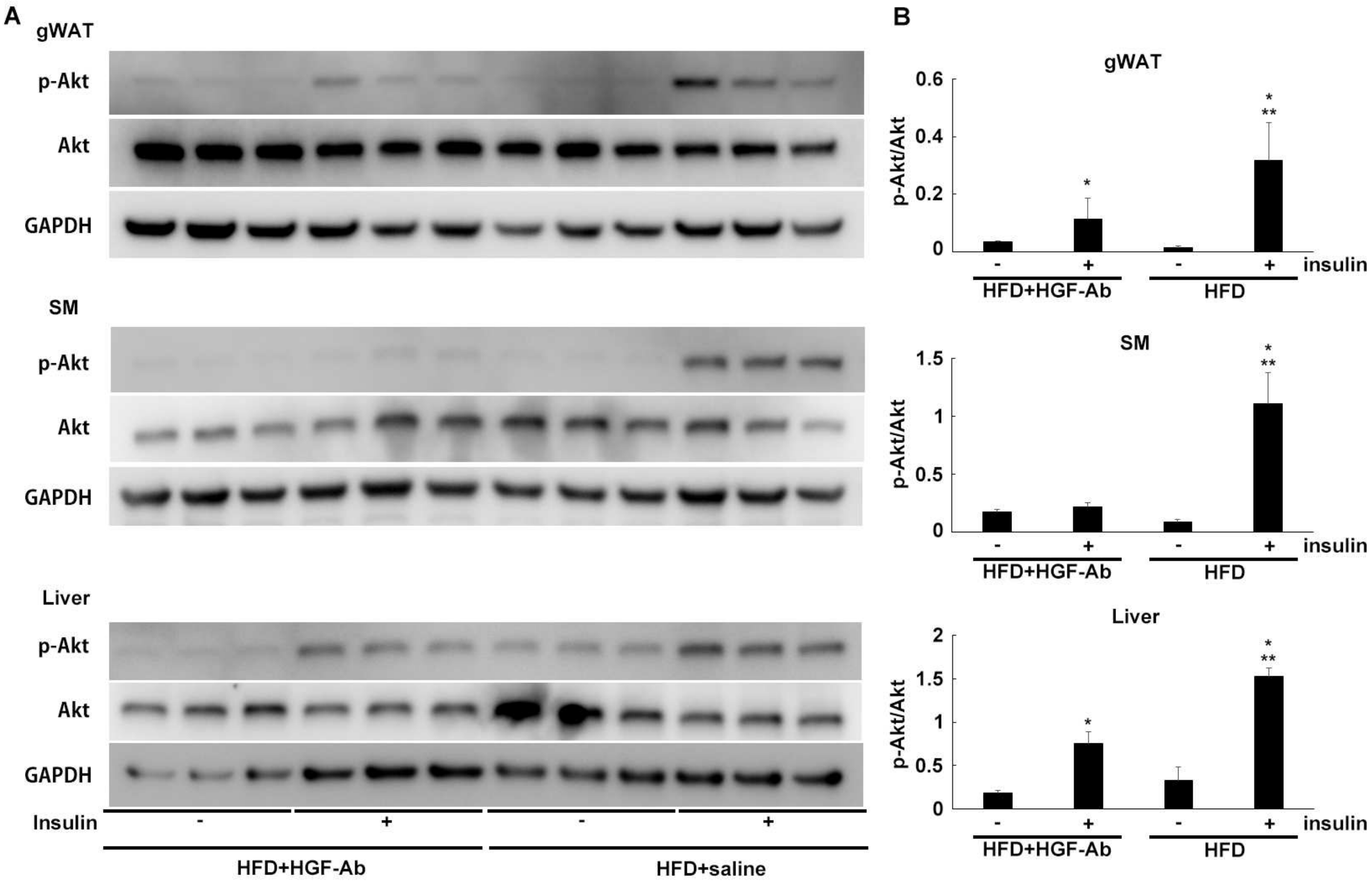

Figure S2 HGF-Ab administration deteriorate insulin resistance induced by HFD.
(A) p-Akt (ser473) levels of the goandal white adipose tissue (gWAT), skeletal muscle (SM), and liver following insulin intraperitoneal injection. N=3.
(B) The ratio of p-Akt/Akt. *P<0.05 vs. corresponding insulin - groups. **P<0.01 vs. HFD+HGF-Ab group. N=3.

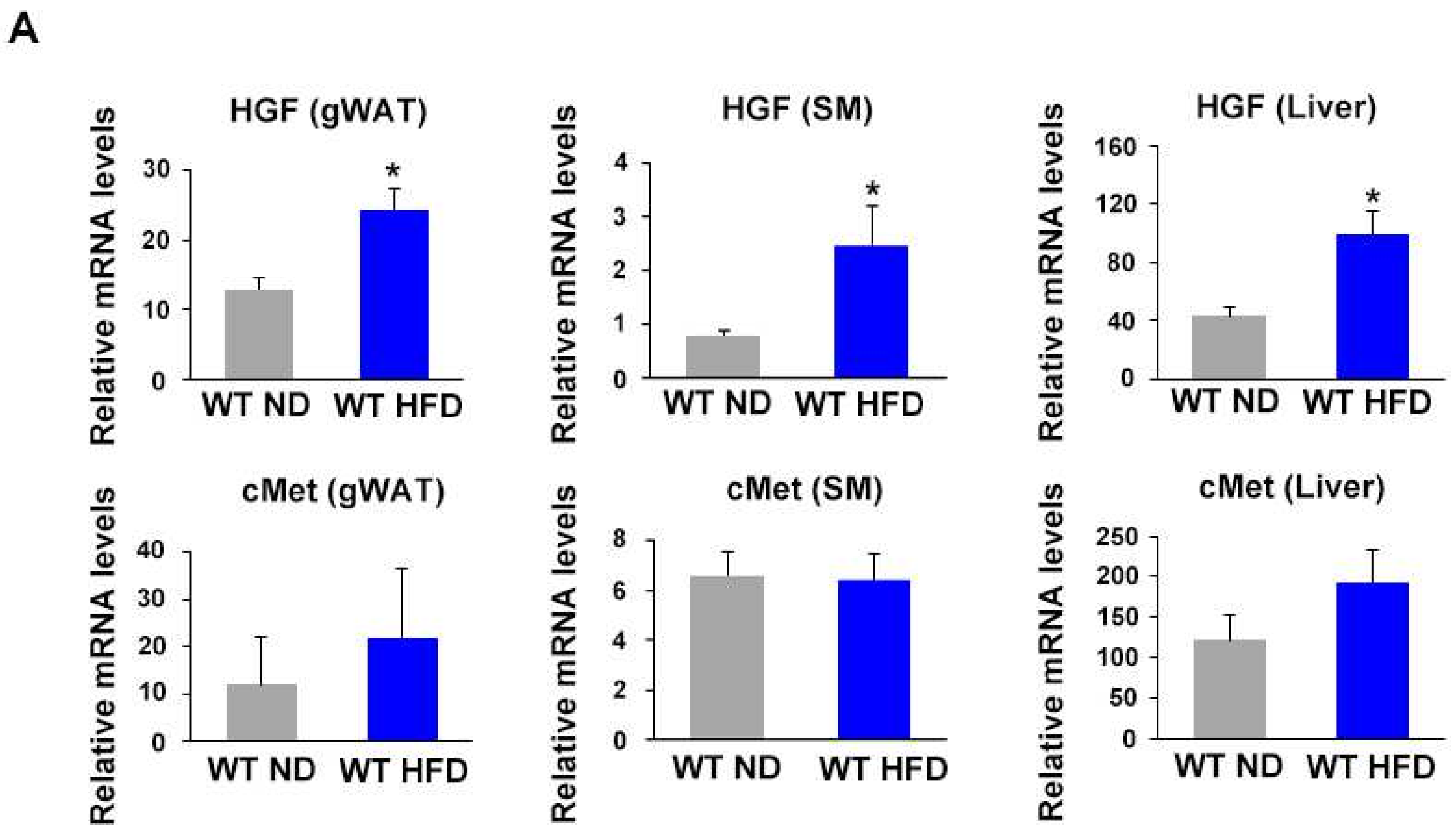

Figure S3 HFD induces HGF and cMet expression.
(A) HGF and cMet mRNA expression of the gonadal white adipose tissue (gWAT), skeletal muscle (SM), and liver in ND or HFD-treated WT mice. N=5-6, *P<0.05 vs. WT ND.

Table S1. Primer sets

Gene	Sense	Antisense
F4/80	CTTTGGCTATGGGCTTCCAGTC	GCAAGGAGGACAGAGTTTATCGTG
MCP-1	ACTGAAGCCAGCTCTCTCTTCCTC	TTCCTTCTTGGGGTCAGCACAGAC
TNF-α	ACGGCATGGATCTCAAAGAC	AGATAGCAAATCGGCTGACG
iNOS	CAGCTGGGCTGTACAAACCTT	CATTGGAAGTGAAGCGTTTCG
IL-1β	CAACCAACAAGTGATATTCTCCATG	GATCCACACTCTCCAGCTGCA
CXCL2	AGTGAACTGCGCTGTCAATG	TCCAGGTCAGTTAGCCTTGC
Adiponectin	ATGGCAGAGATGGCACTCCT	CCTTCAGCTCCTGTCATTCCA
Adiponectin receptor	ACGTTGGAGAGTCATCCCGTAT	CTCTGTGTGGATGCGGAAGAT
HGF	GGCAAGGTGACTTTGAATGA	CACATGGTCCTGATCCAATC
cMet	ACGCAGAAGTTCACCACCAAGTC	CATCACTTCGTACAAGGCGTC
